# Incidence, disease onset and short-term outcome in urea cycle disorders –cross-border surveillance in Germany, Austria and Switzerland

**DOI:** 10.1186/s13023-017-0661-x

**Published:** 2017-06-15

**Authors:** Susanne Nettesheim, Stefan Kölker, Daniela Karall, Johannes Häberle, Roland Posset, Georg F. Hoffmann, Beate Heinrich, Florian Gleich, Sven F. Garbade, On behalf of Arbeitsgemeinschaft für Pädiatrische Stoffwechselstörungen (APS); European registry and network for Intoxication type Metabolic Diseases (E-IMD); Erhebungseinheit für Seltene Pädiatrische Erkrankungen in Deutschland (ESPED); Austrian Metabolic Group; Swiss Paediatric Surveillance Unit (SPSU)

**Affiliations:** 1 Division of Neuropediatrics and Metabolic Medicine, Center for Child and Adolescent Medicine, University Hospital Heidelberg, Im Neuenheimer Feld 430, 69120 Heidelberg, Germany; 2 Medical University of Innsbruck, Clinic for Pediatrics I, Inherited Metabolic Disorders, Innsbruck, Austria; 3 University Children’s Hospital Zurich, Division of Metabolism and Children’s Research Center, Zurich, Switzerland; 4 Erhebungseinheit für Seltene Pädiatrische Erkrankungen in Deutschland, Coordination Center for Clinical Studies, Heinrich Heine University Düsseldorf, Düsseldorf, Germany

**Keywords:** Urea cycle disorder(s), Hyperammonemia, Incidence, Mortality, Newborn screening, Outcome

## Abstract

**Background:**

Urea cycle disorders (UCDs) are a group of rare inherited metabolic disorders. Affected individuals often present with hyperammonemic encephalopathy (HE) and have an increased risk of severe neurologic disease and early death. The study aims to provide epidemiologic data and to describe the disease manifestation and short-term outcome.

**Method:**

Cross-border surveillance of newly diagnosed patients with UCDs - below 16 years of age - was performed from July 2012 to June 2015 in Germany and Austria and from January 2012 to December 2015 in Switzerland. Inquiries were sent monthly to all Pediatric Departments in Germany and Switzerland, and quarterly to the Austrian Metabolic Group. In addition, data were collected via a second source (metabolic laboratories) in all three countries.

**Results:**

Between July 2012 and June 2015, fifty patients (Germany: 39, Austria: 7, Switzerland: 4) with newly diagnosed UCDs were reported and later confirmed resulting in an estimated cumulative incidence of 1 in 51,946 live births. At diagnosis, thirty-nine patients were symptomatic and 11 asymptomatic [10 identified by newborn screening (NBS), 1 by high-risk-family screening (HRF)]. The majority of symptomatic patients (30 of 39 patients) developed HE with (*n* = 25) or without coma (*n* = 5), 28 of them with neonatal onset. Despite emergency treatment 15 of 30 patients with HE already died during the newborn period. Noteworthy, 10 of 11 patients diagnosed by NBS or HRF remained asymptomatic. Comparison with the European registry and network for intoxication type metabolic diseases (E-IMD) demonstrated that cross-national surveillance identified a higher number of clinically severe UCD patients characterized by earlier onset of symptoms, higher peak ammonium concentrations in plasma and higher mortality.

**Conclusion:**

Cross-border surveillance is a powerful tool to identify patients with UCDs demonstrating that (1) the cumulative incidence of UCDs is lower than originally suggested, (2) the mortality rate is still high in patients with neonatal onset of symptoms, and (3) onset type and peak plasma ammonium concentration predict mortality.

## Background

Urea cycle disorders (UCDs) are caused by inherited deficiencies of six enzymes and two transporters that are involved in irreversible detoxification of ammonium to urea. They include deficiencies of N-acetylglutamate synthase (NAGS-D; OMIM #237310), carbamylphosphate synthetase 1 (CPS1-D; OMIM #237300), ornithine transcarbamylase (OTC-D; OMIM #311250), argininosuccinate synthetase (ASS-D; OMIM #215700), argininosuccinate lyase (ASL-D; OMIM #207900), arginase 1 (ARG1-D; OMIM #207800), citrin or aspartate/glutamate carrier (citrin-D, OMIM #603471 and #605814) and the mitochondrial ornithine transporter 1 causing hyperornithinemia-hyperammonemia-homocitrullinuria syndrome (HHH syndrome; OMIM #238970) [1]. Recently, inherited deficiency of carbonic anhydrase VA (OMIM #615751) has been identified as an additional, novel molecular cause of hyperammonemic encephalopathy (HE) in infancy and early childhood showing biochemical overlap with NAGS-D and CPS1-D but also with carboxylases-dependent metabolic disorders [2]. Except for OTC-D, which has an X-linked inheritance, all UCDs are autosomal recessive. UCD patients often present with early onset of symptoms (EO, onset in the first 28 days) during the newborn period, but can present with first symptoms at any age afterwards (late onset, LO). Onset type is correlated with clinical severity and outcome [3–5]. The most severe manifestation is neonatal HE with coma (HE + C); it is associated with a high risk of mortality and, in survivors, of severe neurologic dysfunction and intellectual disability [6–8]. Noteworthy, previous studies from the US and Japan showed higher survival rates than those from Europe [9, 10]. Estimated cumulative incidence of UCDs varies between 1:50,000 [11] and 1:8000 live births [12]. OTC-D, ASS-D and ASL-D are the most common UCDs representing about 90% [13]. Most likely the true incidence is higher since some cases may remain undiagnosed: Patients with EO are sometimes misdiagnosed as neonatal infections, whereas UCD patients with LO may be missed due to their variable phenotypic spectrum including hepatological, gastrointestinal, neurological and psychiatric disease manifestations [14–17]. Guidelines for diagnosis and management of UCDs have been published [1]. Maintenance treatment of UCDs is based on a low protein diet, supplementation with essential amino acids, citrulline and/or arginine, vitamins and minerals, and application of nitrogen scavenger drugs (sodium benzoate, sodium and glycerol phenylbutyrate) as well as carglumic acid (for NAGS-D and partially responsive CPS1-D). During HE, treatment is intensified stepwise by implementation of intravenous nitrogen scavenging therapy and extracorporeal detoxification [1, 18, 19]. Liver transplantation is a curative therapy for UCDs. Best outcome is achieved if liver transplantation is carried out between age 3–12 months and before irreversible brain damage manifests [1, 20, 21]. Hepatocyte transplantation is an experimental therapy which can be used as a bridging procedure. Since transplanted cells are short-lived, the need for liver transplantation has not been averted by this approach [22–24]. One of the major concerns is timely identification and undelayed start of severity-adjusted therapy. For this purpose, some UCDs – mostly ASS-D and ASL-D – have been included in newborn screening (NBS) programmes in a few countries [13, 25]. Although preliminary results are promising for LO patients, effectiveness remains to be demonstrated for EO patients [5]. The study aims to provide epidemiologic data and to describe the severity of disease manifestation, mortality and short-term outcome in UCD patients in Germany, Austria and Switzerland.

## Methods

### Active surveillance of symptomatic individuals with urea cycle disorders

Newly diagnosed individuals with UCDs - below 16 years of age - were included in the cross-border nationwide surveillance between 1st July 2012 and 30th June 2015. In Germany, inquiries were sent monthly by German Pediatric Surveillance Unit (ESPED) to all Departments of Pediatrics (source A) and quarterly by University Hospital Heidelberg to all specialized metabolic laboratories (source B). In Austria, inquiries were sent quarterly by one of the authors (DK) to the Austrian Metabolic Group (source A) which includes pediatric metabolic physicians, internists, clinical chemists, clinical geneticists, and personnel from the Austrian NBS program and specialized metabolic laboratories (source B). In Switzerland, inquiries were sent monthly by *Swiss Paediatric Surveillance Unit* (SPSU) to all pediatric hospitals (source A) and quarterly by University Children’s Hospital Zurich to all specialized metabolic laboratories (source B). Newly diagnosed individuals with UCDs were included into an MS Access database using a standardized questionnaire with comprehensive information on the diagnostic process, disease subtype, date of birth (month and year), description of the first clinical presentation, start and mode of specific metabolic treatment, characteristic laboratory findings such as peak plasma ammonium, plasma amino acids and urinary orotic acid, and short-term clinical outcome. Information about gestational age and complications in pregnancy were not captured. The return rate of questionnaires was 100% in Switzerland and Auatria, and 98% in Germany. Seven individuals were excluded from the study in Germany, four with suspected but unconfirmed UCDs and three due to disease manifestation before the start of the study. Three Austrian UCD patients diagnosed outside the study period or being older than 16 years of age were also excluded. In Switzerland, 6 patients were excluded due to criteria not met (*n* = 3), double reporting (*n* = 1), reporting of a known patient (*n* = 1), and yet unconfirmed UCD with investigations ongoing (*n* = 1). False positive and double reports were excluded from the analysis.

### Comparison between this study and the E-IMD sample

Case mix, onset type, peak plasma ammonium concentrations and clinical symptoms during the initial clinical manifestation, and mortality were compared between this study and a previously published sample of 456 UCD patients [5].

### Statistical analysis

Incidence of definite cases with UCDs were estimated from the number of live births in Germany (*n* = 2,102,583), Austria (*n* = 242,351) and Switzerland (*n* = 252,380) during the study period. Cases reported could be born before the start of the common study period (1st July 2012) and could therefore belong to earlier birth cohorts, whereas affected individuals born between 1st July 2012 to 30th June 2015 could have remained asymptomatic during the surveillance period. Furthermore, capture-recapture analysis was used as previously described [26, 27]. This methodology was designed to estimate population sizes on the basis of the proportion of individuals (re)captured by two or more sources. The Chapman estimate of the true number of cases has been calculated as

$$ \boldsymbol{N}=\frac{\left(\boldsymbol{na}+1\right)\left(\boldsymbol{nb}+1\right)}{\left(\boldsymbol{na}\boldsymbol{b}+1\right)}-1 $$

with *n*_*a*_ and *n*_*b*_ denoting the number of individuals captured in data sources A and B, respectively, and *n*_*ab*_ representing individuals captured in both sources. Categorical and count data were analyzed with log-linear models. Mann-Whitney U-test was computed to detect differences in central tendency for two groups. Differences in central tendency between three or more groups were evaluated with Aligned Rank Transform (ART) for nonparametric factorial analyses. ART, a conventional analysis of variance (ANOVA), was used for computation after the dependent variable was “aligned” for each effect before ranked [28]. The R environment for statistical computing (2016) was used for statistical and graphical computations, ARTool package for R [28] was used to calculate ART ANOVA. Post hoc contrasts were carried out with “least square means” package for R [29]. Log-linear models were estimated using vcd-package for R [30].

## Results

### Description of the study sample

Cross-border surveillance in Germany, Austria, and Switzerland over three years (1st July 2012 to 30th June 2015) identified a total of 50 patients (Germany: 39 individuals, Austria: 7, Switzerland: 4) with newly diagnosed UCDs (Table 1). All identified patients lived in Germany, Austria or Switzerland. UCD patients from other countries were not included in this study. Tracking between source A and B was possible in Germany, so capture-recapture analysis was computed only for German cases. In Germany, 35 patients were reported via source A, 25 via source B and 21 via both sources, all in all 39 patients. Since five individuals were exclusively reported by metabolic laboratories, we did not receive clinical information about these individuals. In Austria and Switzerland, UCD patients were exclusively reported via source A. Reported individuals with UCDs were born between June 1998 and April 2015, 34 of them being males and 16 females. The discrepancy in sex distribution was expected. It is explained by the high frequency of OTC-D (*n* = 22 of 50 reported UCD patients) in the study sample and the higher frequency of symptomatic male (mOTC-D, *n* = 18) compared to female OTC-D (fOTC-D, *n* = 4) patients due to X inheritance. All other UCDs were less frequently found (CPS1-D: *n* = 8, ASS-D: *n* = 10, ASL-D: *n* = 7, ARG1-D: *n* = 1, HHH syndrome: *n* = 1, citrin-D: *n* = 1). Individuals with NAGS-D were not reported. Suspected diagnoses were confirmed biochemically (*n* = 48) and/or by enzyme (*n* = 2) and/or mutation analysis (*n* = 34). Twenty of the UCD patients had family members with the same disease (18 patients with one, and either one patient with two or four affected family members). Thirteen family members had OTC-D, six had ASS-D, four had CPS1-D, and one had HHH syndrome. The majority of individuals with UCDs were diagnosed after the manifestation of symptoms (*n* = 39), whereas in some the diagnosis was made before disease onset, either by NBS (*n* = 10) or by HRF (*n* = 1), i.e. in a family with a previously identified index patient. Patients identified by NBS were all from Germany. Although the national German screening panel does not include UCDs, pilot studies for UCDs are ongoing in some NBS laboratories. Symptomatic UCD patients usually presented with first symptoms during the newborn period (EO group: *n* = 28; median age at disease onset: 2 days, range: 1–7 days), whereas seven LO patients presented at a median age of 1089 days (range: 303–4018 days). Overall, diagnosis was made at a median age of 3 days (range: 2–5844 days), however, with a marked difference between the EO and LO groups [median age (range) at diagnosis, EO: 3 (2–8) days; LO: 2178 (696–5844) days] and between single UCDs (minimum: 2 days in CPS1-D, maximum: 5844 days in fOTC-D). Furthermore, the diagnostic delay, i.e. the time interval between the manifestation of first symptoms and diagnosis, markedly differed between EO (median 1 day delay) and LO (median: 1089 days delay) groups.

**Table 1:** Reported individuals with urea cycle disorders

Country	Source	Reports (total)	Reports (eligible)^a^
Germany	A or B	58^b^	39
	A	52	35
	B	27	25
	A and B	21	21
Austria	A or B	10^b^	7
Switzerland	A or B	11^b^	4
All countries	A or B	79^b^	50

^a^Excluding false positive (unconfirmed diagnosis, onset of symptoms before start of study, age at diagnosis above 16 years of age) and double reports

^b^Number of patients reported via at least one source

### Clinical presentation and short-term outcome

A broad spectrum of clinical symptoms was found during the initial presentation. These include HE (with or w/o coma) in 30 patients, neurological (seizures/epilepsy: 16 patients, delayed motor development: *n* = 3, delayed cognitive development: *n* = 3, movement disorders: *n* = 1, behavioral abnormalities: *n* = 4) and gastrointestinal symptoms (recurrent vomiting: *n* = 8, protein aversion: *n* = 4, failure to thrive: *n* = 1) as well as acute liver failure (*n* = 5). Ten individuals with OTC-D (*n* = 1), ASS-D (*n* = 4), ASL-D (*n* = 3), ARG1-D (*n* = 1), and citrin-D (*n* = 1) were identified by NBS. One individual identified by NBS developed symptoms on fifth day of life. Whereas neurological symptoms were found across different UCDs, acute liver failure was exclusively observed in patients with mOTC-D (*n* = 3) and fOTC-D (*n* = 2), protein aversion mostly in fOTC-D (*n* = 3), and recurrent vomiting mostly in mOTC-D (*n* = 3) and fOTC-D (*n* = 3). Delayed motor and cognitive development, movement disorder, protein aversion, and failure to thrive were exclusively found in the LO group, and behavioral abnormalities (EO: 1/28, LO: 3/7 patients), recurrent vomiting (EO: 4/28, LO: 4/7 patients) and acute liver failure (EO: 3/28, LO: 2/7 patients) were more frequent in the LO than in the EO group. HE was reported in 30 individuals with UCDs, 25 of them being comatose (HE + C) on admission (Table 2). It was most commonly found in the EO group (HE total: 27/28, HE + C: 24/27 patients) and less often in the LO group (HE total: 3/7, HE + C: 1/3 patients). Highest frequency and most severe HE was found in CPS1-D (HE total: 7/8, HE + C: 6/7 patients) and mOTC-D (HE total: 15/17, HE + C: 13/15 patients).

**Table 2:** Frequency of hyperammonemic encephalopathy (initial presentation)

Disease name	Patients (*n*)	HE total (*n*)	HE + C (*n*)
CPS1-D	8	7	6
mOTC-D	17	15	13
fOTC-D	5	1	0
ASS-D	10	3	3
ASL-D	7	4	3
ARG1-D	1	0	0
HHH syndrome	1	0	0
Citrin-D	1	0	0
Total	50	30	25

*HE* hyperammonemic encephalopathy, *HE + C* HE with coma. In a single patient with CPS1-D the manifestation of HE remained unknown

Clinical severity was paralleled by peak plasma ammonium concentrations: Highest peak plasma ammonium concentrations were reported for CPS1-D and mOTC-D. Initial ammonium concentrations were much higher in the EO group than in the LO group and higher in the HE + C group compared to HE without coma and asymptomatic (AS) individuals (Table 3).

**Table 3:** Peak plasma ammonium concentration (before treatment)

	Patients (*n*)	Median (Range) (μmol/L)
Disease name		
CPS1-D	8	2333 (350–5400)
mOTC-D	17	1714 (110–8160)
fOTC-D	5	247 (73–498)
ASS-D	10	422 (40–1266)
ASL-D	7	320 (36–1471)
ARG1-D	1	63 (n/a)
HHH syndrome	1	60 (n/a)
Citrin-D	1	44 (n/a)
Total	50	935 (36–8160)
Onset type^a^		
EO	28	1622 (199–8160)
LO	7	247 (60–498)
Asymptomatic	10	68 (36–110)
Unknown^a^	5	n/a
Initial hyperammonemia		
HE	5	320 (150–1383)
HE + C	25	1945 (298–8160)

Peak plasma ammonia levels are different between EO and LO groups (U-test, *p* < 0.0001) as well as between EO or LO and asymptomatic patients, respectively (U-test, *p* < 0.001). Note that plasma ammonium concentrations are age-dependent: Newborns (arterial cord blood), 50–159 μmol/L; infants and children, 24–48 μmol/l; adults, 11–55 μmol/L (adapted from [38]). Peak plasma ammonia levels differed between groups AS, HE and HE + C [ART ANOVA: F(2,37) = 36.63; *p* < 0.001]. Contrasts with Tukey *p*-value adjustment revealed significant differences between all three groups: AS vs. HE (*p* = 0.024), AS vs. HE + C (*p* < 0.001) and HE vs. HE + C (*p* = 0.005). EO, early onset; HE, hyperammonemic encephalopathy; HE + C, HE with coma; LO, late onset; n/a, not applicable

^a^Five patients in whom clinical information was not available since they have been exclusively reported by metabolic laboratories

As a consequence of severe HE, the majority of patients from the EO group (21/28) required hemodialysis/hemofiltration (20/21) or peritoneal dialysis (1/21), whereas only one individual of the LO group required extracorporeal detoxification (1/7 patients). Scavenger therapy was applied to all individuals of the EO group (28/28 patients) and to all except two patients from the LO group (5/7 patients). Sodium benzoate was more frequently administered (EO: 28/28, LO: 5/7 patients) than sodium phenylbutyrate or sodium phenylacetate (EO: 14/28, LO: 3/7 patients). Probatory therapy with carglumic acid was exclusively made in the EO group (13/28 patients). Despite emergency therapy 15 of 30 UCD patients (50%) with HE died during or shortly after the clinical manifestation. Brain edema/herniation (*n* = 7) and multi-organ failure (*n* = 5) were the most frequently reported causes of death. The mortality rate was highest in patients with mOTC-D (mortality rate: 10/17 patients = 59%) and CPS1-D (4/8 patients = 50%). All deceased patients belonged to the EO group (mortality rate: 15/28 EO patients = 54%) highlighting that the age of disease onset, severity of clinical symptoms and hyperammonemia predicted mortality. Noteworthy, nine of ten individuals with UCDs who were identified by NBS and one patient with HRF remained asymptomatic until the end of the observation period.

### Comparison between this study and the E-IMD sample

Cross-border national surveillance of UCDs in Germany, Austria and Switzerland demonstrated a high frequency of EO patients (28/35 = 80% of symptomatic patients with known onset type), and a clear correlation between the severity of the initial manifestation (HE, coma, peak plasma ammonium concentration) and mortality. The proportion of EO patients in our sample was much higher than in the recently published European UCD sample of the E-IMD patient registry (123/456 = 27% of symptomatic patients) [5]. The design of both observational studies is different: (1) UCD patients in the E-IMD registry are exclusively registered by specialized metabolic centres, whereas the sample of the present study is based on nationwide surveillances in Germany, Austria and Switzerland; (2) the E-IMD registry includes UCD patients of any age of diagnosis, whereas this study only includes patients in whom diagnosis has been made before age 16 years (in E-IMD, 379/456 = 83% patients were diagnosed below age 16 years). To compare whether differences between the E-IMD study and our study were exclusively explained by differences in the study design or by other factors, we first compared the case mix of both samples (Table 4). Individuals with CPS1-D were more frequent than expected and those with fOTC-D less frequent than expected in this study compared to the E-IMD sample (using Pearson residuals, *p* = 0.004). Next, we calculated whether these differences were explained by the age limit in this study (i.e. age of diagnosis below 16 years). Omitting 77 UCD patients diagnosed after age 16 years from the E-IMD sample did in fact eliminate the above mentioned conditions for fOTC patients, however CPS1-D patients still remained more frequent than expected in this study. Furthermore, median age of diagnosis was much lower [this study: 3 days, E-IMD with age of diagnosis below 16 years: 210 days (W = 12,395, *p* < 0.001, U-test with continuity correction)], whereas median peak plasma ammonium concentration [this study: 968 μmol/L, E-IMD: 372 μmol/L; W = 4013, *p* = 0.0495, U-test with continuity correction)] and mortality rate were higher [this study: 15/49 = 31%, E-IMD: 12/379 = 3%; *p* < 0.001)] in this study than in the E-IMD sample. This demonstrates that the cut-off for age of diagnosis (i.e., 16 years) used in this study can only explain the less frequent than expected fOTC patients, but not the higher clinical severity and mortality of UCD patients in the present study.

**Table 4:** Case mix of this study and the E-IMD study

Disease name	E-IMD study Patients (*n*, %)	E-IMD study, age of diagnosis below 16 years^a^ Patients (*n*, %)	This study Patients (*n*, %)
NAGS-D	9 (2%)	7 (2%)	0
CPS1-D	21 (5%)	20 (5%)	8 (16%)
mOTC-D	109 (24%)	95 (25%)	17 (34%)
fOTC-D	146 (32%)	98 (26%)	5 (10%)
ASS-D	87 (19%)	82 (22%)	10 (20%)
ASL-D	61 (13%)	56 (15%)	7 (14%)
ARG1-D	12 (3%)	12 (3%)	1 (2%)
HHH syndrome	11 (2%)	9 (2%)	1 (2%)
Citrin-D^b^	n/a	n/a	1 (2%)
Total	456 (100%)	379 (100%)	50 (100%)

^a^Using the same age cut-off as in the present study

^b^Citrin deficiency was excluded from the analysis since this disease is not included in the E-IMD study [5]

### Epidemiology

Cumulative estimated incidence of UCDs was calculated based on the number of newly diagnosed UCD patients (Germany: 39, Austria: 7, Switzerland: 4) and the number of live births in Germany (*n* = 2,102,583), Austria (*n* = 242,351) and Switzerland (*n* = 252,380) during the common study period from 1st July 2012 to 30th June 2015. Accordingly, estimated incidences were 1 in 53,912 (Germany; 95% CI 53,840–53,8985), 34,622 (Austria; 95% CI 34,483–34,760) and 63,095 (Switzerland; 95% CI 62,849–63,341), with a cumulative estimated incidence of 1 in 51,946 (95% CI 51,883–52,009) live births for all countries. Since affected individuals were reported via two sources in Germany, we also estimated the incidence of the German sample using the capture-recapture analysis. This approach revealed a slightly higher incidence of 1 in 50,609 live births. The estimated overall cumulative incidence is under-estimated due to exclusion of UCD patients diagnosed after the age of 16 years. Assuming a similar proportion of UCD patients diagnosed after the age of 16 years as in the E-IMD sample (i.e. 77/456 = 17% of UCD patients) the estimated incidence would increase to 1 in 38,406 live births in the three countries, still missing patients who remain un−/misdiagnosed.

## Discussion

The major results of this cross-border study from Germany, Austria and Switzerland are that (1) the estimated cumulative incidence of UCD patients under 16 years of age is about 1 in 52,000 live births, (2) registries and observational studies focusing on the follow-up of surviving patients are likely to underestimate the number of UCD patients with a severe phenotype, (3) despite increased awareness for UCDs, improved NICU care, and evidence-based guidelines, the mortality rate of EO patients is still high, in particular for CPS1-D and mOTC-D, and (4) UCD patients identified by NBS had a favourable short-term outcome.

### Incidence

This study places the estimated incidence or UCDs below 16 years of age at about 1 in 52,000 live births in Germany, Austria and Switzerland ranging from about 1 in 63,000 (Switzerland) to 1 in 35,000 live births (Austria). Assuming that the same cumulative incidence is found in the 28 EU Member States, at least 100 new UCD patients are to be expected each year (2015: 5,091,295 live births, http://www.ec.europa.eu/eurostat. Similar estimated incidences have been reported in three previous publications from the US (1 in 35,000 live births; [13]), Japan (1 in 50,000 live births; [11]) and Finland (1 in 39,000 live births; [31]). A previous report from the US with a cumulative incidence of 1 in 8000 live births [12], however, is likely to over-estimate the total incidence of UCD patients. Although our study provides the first robust estimation of incidence for UCDs in Germany, Austria and Switzerland, we assume that the true incidence is under-estimated for the following reasons: First, the number of UCD patients who have not been reported or in whom the diagnosis has been missed is unknown. Second, this study has excluded UCD patients diagnosed after age 16 years, probably around 20%. The risk of missing UCD patients is still a relevant source of bias since patients with a fulminant phenotype might die early and undiagnosed and later manifestations can be unspecific. This notion is supported by the fact that 15 of 30 patients with HE in our study died shortly after the manifestation of symptoms. A recent meta-analysis evaluating 29 studies published between 1978 and 2014 with 1542 participants in the US, Japan and Europe confirms the overall high risk of neonatal death in UCD patients with EO disease manifestation, except for ASL deficiency [7].

### Clinical presentation and mortality

The clinical spectrum of initial presentations of UCD patients in this study is broad including overwhelming disease, neurological, gastrointestinal or hepatic symptoms. This is in line with previous studies from the US and Europe [4, 17]. The broad range of differential diagnoses with above mentioned clinical symptoms increases the risk of missing the diagnosis. A limitation of this study is the short observation period which may explain why specific symptoms such as delayed motor and cognitive development and movement disorder were exclusively found in the LO group of patients.

Natural history, clinical outcome and survival of UCD patients differ depending on residual enzyme activity, gender (e.g. OTC-D), peak plasma ammonium concentrations, duration of hyperammonemia, and the age of first symptoms [3–5, 7–9, 15, 17, 18]. Furthermore, clinical outcome depends on local experience and clinical skills of multiprofessional teams carrying for UCD patients [1]. Overall, EO patients, in particular mOTC-D, showed the highest mortality and the poorest neurological outcome among those who survived. However, more recent studies have identified an unexpectedly high number of LO patients and of non-neurological disease manifestations [14–16, 32, 33]. Furthermore, the case mix of onset types differed in study samples worldwide [7]. Whether this reflects true differences between populations or selection bias has remained unclear. The present study speaks in favour of patient registries and longitudinal observational studies particularly of UCD patients with LO and a mild disease course. This is supported by the fact that a higher number of clinically severe EO patients, higher peak plasma ammonium concentrations and higher mortality were identified by the recent cross-border surveillance than in the E-IMD patient registry. Furthermore, our study challenges the recently published notion that survival in UCD patients has improved over the last decades [10, 34]. In contrast, our study demonstrates a continuing high mortality rate of EO patients, in line with a recent meta-analysis [7], and an observation study from Europe [8] We cannot exclude, however, that the mentioned differences between these studies reflect current differences in diagnostic strategies, management and care between Europe, Japan and the US.

### Newborn screening programme for urea cycle disorders?

In Europe, UCDs are rarely – however, ASS-D and ASL-D occasionally – included into national NBS programmes [25], but are commonly included in the US [13]. The clinical benefit of this diagnostic intervention for UCD patients is still unclear. However, a trend towards improved neurological long-term outcome for neonatally screened patients with ASS-D, ASL-D and ARG1-D has been demonstrated recently [5]. Furthermore, NBS may identify individuals with benign phenotypes such as untreated ASL-D individuals with normal intellectual and psychomotor development without any intervention [35–37]. Noteworthy, 10 of 11 UCD patients identified neonatally by NBS or HRF in this study remained asymptomatic during the short observation period highlighting a potentially positive effect of NBS for UCD patients. However, since the design of this study does not allow long-term follow-up and careful evaluation of the natural history in this subgroup, these studies still remain to be performed. Furthermore, it should be considered that the time window to identify newborns before the onset of first symptoms is often narrow so that the impact of NBS on the outcome critically relies on the process quality of NBS programs such as the time between taking the NBS samples and having the NBS results. More systematic work and long-term observational studies are required to elucidate whether NBS programmes can reduce mortality and improve the long-term neurological and intellectual outcome of UCD patients. Most importantly, we also need improved therapeutic approaches.

## Conclusion

The cumulative incidence of UCDs in this study is in a similar range as incidences recently reported for the US, Japan and Finland [11, 13, 31] but significantly lower than previously suggested [12]. This should be taken into consideration for the design of future clinical trials and care programmes. Despite increasing awareness, beginning harmonization of management and care in Europe according to a recently published guideline [1], and improved emergency and NICU treatment, the mortality rate of UCD patients with and EO disease course is still very high in Germany, Austria and Switzerland. This highlights that more efforts are required to identify affected individuals with UCDs as early and as reliable as possible. Although new therapies have been introduced over the years, improvement of clinical outcome and mortality might simply reflect discrepancies in the case mixes of the different study samples investigated.

## Abbreviations

ANOVA: Analysis of variance
ARG1-D: Arginase 1 deficiency (synonym, argininemia)
ART: Aligned rank transform
AS: Asymptomatic
ASL-D: argininosuccinate lyase deficiency
ASS-D: Argininosuccinate synthetase 1 deficiency (synonym, citrullinemia type 1)
CI: Confidence interval
Citrin-D: Citrin deficiency (synonym, citrullinemia type 2)
CPS1-D: Carbamylphosphate synthetase 1 deficiency
E-IMD: European registry and network for Intoxication type Metabolic Diseases
EO: Early onset of symptoms (i.e., during the newborn period)
ESPED: German pediatric surveillance unit
F: Female
HE: Hyperammonemic encephalopathy
HE + C: Hyperammonemic encephalopathy with coma
HHH syndrome: Hyperornithinemia-hyperammonemia-homocitrullinuria syndrome
HRF: High-risk family screening (i.e. family with a previously identified index patient)
LO: Late onset of symptoms (i.e., after the newborn period)
M: Male
NAGS-D: N-acetylglutamate synthase deficiency
NBS: Newborn screening
OTC-D: Ornithine transcarbamylase deficiency
SPSU: Swiss paediatric surveillance unit
UCD(s): Urea cycle disorder(s)
